# Effect of dietary biochanin A on lactation performance, antioxidant capacity, rumen fermentation and rumen microbiome of dairy goat

**DOI:** 10.3389/fmicb.2023.1101849

**Published:** 2023-02-06

**Authors:** Qingbiao Xu, Yanjun Li, Wenjuan Du, Nan Zheng, Jiaqi Wang, Shengguo Zhao

**Affiliations:** ^1^State Key Laboratory of Animal Nutrition, Institute of Animal Sciences, Chinese Academy of Agricultural Sciences, Beijing, China; ^2^College of Animal Sciences and Technology, Huazhong Agricultural University, Wuhan, China; ^3^MoE Key Laboratory of Molecular Animal Nutrition, College of Animal Sciences, Zhejiang University, Hangzhou, China

**Keywords:** antioxidant capacity, biochanin A, dairy goat, lactation performance, rumen microbiome, ruminal fermentation

## Abstract

Biochanin A (BCA), an isoflavone phytoestrogen, is a secondary metabolite produced mainly in leguminous plants. The objective of this study was to evaluate the effect of BCA on lactation performance, nitrogen metabolism, and the health of dairy goat. Thirty mid-lactation Saanen dairy goats were divided into three groups randomly: control, 2 g/d BCA group, and 6 g/d BCA group. After 36 days of feeding, 30 dairy goats were transferred to individual metabolic cages. Subsequently, milk yield, feed intake, total feces, and urine excretion were recorded and samples were collected continuously for 3 days. Blood and ruminal fluid samples were collected over the subsequent 4 days. Milk yield, milk protein, fat content, and the feed conversion ratio of dairy goat were significantly increased by the BCA treatment. The levels of serum 17β-estradiol, growth hormone, insulin-like growth factor 1, glutathione peroxidase activity, and total antioxidant capacity were also increased significantly by BCA, indicating that BCA enhanced the antioxidant capacity of dairy goat. Amino acid degradation was significantly inhibited, while the ammonia nitrogen content was reduced significantly by BCA. Total volatile fatty acids was significantly increased by BCA supplementation. In addition, the relative abundance of *Verrucomicrobiota* was decreased significantly. However, the growth of nitrogen metabolism and cellulolytic bacteria was significantly increased under BCA treatment, including *Prevotella sp*., *Treponema sp.*, *Ruminococcus flavefaciens*, and *Ruminobacter amylophilus*. In conclusion, supplementation with BCA improved the milk production performance, nitrogen metabolism, rumen fermentation and antioxidant capacity, and regulated the rumen microbiome of dairy goat.

## Introduction

Biochanin A (BCA), an isoflavone phytoestrogen with various functions (Wenjin et al., 2021), is a secondary metabolite produced mainly in leguminous plants such as red clover and chickpea (Křížová et al., 2019). The BCA content in red clover (*Trifolium pratense L.*) leaves during the flowering stage ranges from 4.57 to 6.86 mg/g dry matter (DM), with an average concentration of 5.76 mg/g DM, which accounts for approximately 40% of the total isoflavones (Lemeziene et al., 2015). Chickpea (*Cicer arietinum L.*) sprouts contain lower concentration of BCA than red clover, with a maximum concentration of 2.34 mg/g DM (Gao et al., 2015). Sonication of peanuts (*Arachis hypogaea*) in 80% aqueous ethanol yielded a BCA content of 0.964 mg/100 g DM (Chukwumah et al., 2009). Young plants of *Astragalus glycyphyllos L.* and *Astragalus cicer L.* had BCA contents of 8.81 and 11.4 mg/100 g DM, respectively (Butkute et al., 2018). Other natural sources of BCA include alfalfa (Kagan et al., 2015) and soybeans (Hu et al., 2021). Recent studies have demonstrated that BCA possesses numerous biological properties, such as anti-inflammatory, anti-microbial, and antioxidant activities (Jalaludeen et al., 2016; Sadri et al., 2017). Furthermore, BCA has been shown to be a potential drug for protection against osteoporosis (Lee and Choi, 2005), prevention and treatment of atherosclerotic cardiovascular disease (Yu et al., 2020), and alleviation of symptoms in postmenopausal women (Galal et al., 2018). Therefore, BCA is an important source of novel health foods and natural products.

Hyper-ammonia-producing bacteria (HAB) play a vital role in amino acid (AA) deamination in the rumen (Flythe and Kagan, 2010). *In vitro*, BCA was shown to possess antimicrobial activity against HAB isolated from the rumen fluid of bovines and caprines; however, it has to act synergistically with other heat-stable antibacterial compounds to inhibit HAB growth (Flythe and Kagan, 2010; Flythe et al., 2013). In addition, BCA exhibited selective antimicrobial activity against amylolytic and cellulolytic bacteria, and increased *Lactobacilli*, decreased gram-positive *cocci*, and directly inhibited the growth of *Fibrobacter succinogenes S85*, *Ruminococcus flavefaciens 8*, and *Ruminococcus albus 8* (Harlow et al., 2017b,^2018^). These results were attributed to the enhancement of the activities of native rumen antimicrobials (e.g., bacteriocins) by BCA (Flythe et al., 2013; Harlow et al., 2017b). Studies have also shown that BCA can inhibit lactic acid production and increase lactate-utilizing bacteria and lactic acid metabolism, thereby ameliorating pH decline, promoting rumen fermentation, and increasing the concentrations of acetate, propionate, and total volatile fatty acids (VFAs) (Harlow et al., 2017b,^2018^). Furthermore, BCA could be a substitute for antibiotics, such as monensin, for reducing rumen acidosis (Harlow et al., 2017c,^2021^). Supplementing steers fed dried distillers’ grains (DDG) with BCA improved crude protein (CP) digestibility of DDG and increased average daily gain, as the addition of BCA decreased ammonia production and improved the quality of absorbed protein by inhibiting HAB (Flythe et al., 2013; Harlow et al., 2017a). Similar results were observed with red clover whereby the average daily gain depended on the interaction between BCA and other isoflavones (Harlow et al., 2020).

In our lab, it was observed that BCA inhibited the activity of proteolytic and ureolytic bacteria and improved the efficiency of microbial protein synthesis (Liu et al., 2020). Therefore, BCA is hypothesized to be a potential novel urease inhibitor. However, to date, there is limited information on the *in vivo* effects of BCA on dairy livestock. Therefore, the objective of this study was to evaluate the effects of BCA on production performance, health, and rumen microbiome of dairy goats.

## Materials and methods

### Animal management

Animal experiments were approved by the Animal Care and Use Committee of the Institute of Animal Science of the Chinese Academy of Agricultural Sciences (approval no. IAS2020-97). Thirty mid-lactation health Saanen dairy goats (body weights of 63.5 ± 5.6 kg at 147 ± 3 d of lactation) from Weihe Dairy Farm (Qiangyang, China) were randomly divided into three groups (*n* = 10): basal diet group (control), basal diet with 2 g/d BCA per goat group (BL), and basal diet with 6 g/d BCA per goat group (BH). To make sure the 6 g/d group indeed ingested 6 g/d BCA a day per goat, BCA was mixed with a small amount of basic diet and fed to each goat separately every morning. After each goat had finished all the basic diet with BCA, the left diet would be given to them. The doses used were based on previous report (Harlow et al., 2017c). The duration of the experiment was 50 d in autumn (September–November), with 43 d permitted for diet acclimatization and 7 d allocated for sample collection. The dairy goats were placed in individual metabolic cages on day 36 of the acclimatization period. The goats were fed, *ad libitum*, a basal diet and total mixed diet (TMR), and were allowed approximately 5% orts twice a day at 08:00 and 14:30. Dairy goats were milked twice daily, at 07:00 and 18:00. The dietary ingredients (% DM) and chemical composition (% DM) of TMR are shown in Table 1.

**TABLE 1:** Ingredient and chemical composition of total mixed diet fed to dairy goats.

Ingredient composition	% of DM	Chemical composition	% of DM
Oat grass	11.42	NE_L_^2^ (Mcal/kg)	1.92
Alfalfa hay	30.06	DM	80.76
Steam-flaked corn	13.49	ADF	28.77
Soybean meal	12.13	NDF	48.70
Cottonseed meal	1.49	CP	16.83
Dried distiller’s grains	1.49	Ether extract	2.52
Bran	6.02	–	–
Wheat shorts	0.60	–	–
Preminx^1^	1.49	–	–
Sprayed corn bran	1.50	–	–
Corn gluten meal	0.84	–	–
Cron bran	6.01	–	–
Yeast	3.84	–	–
Corn silage	8.97	–	–
NaHCO_3_	0.21	–	–
Mycotoxin adsorbent	0.19	–	–
Urea	0.25	–	–

^1^The premix provided the following per kg of diets: vitamin A 650 K international unit (IU), vitamin D 350 K IU, vitamin E 4,000 IU, Zn 2,500 mg, Cu 200 mg, Mn 1,200 mg.

^2^NE_L_ = lactation net energy, as calculated with reference to nutrient requirements of dairy cattle (NRC) (2007) nutrient requirements of goats.

### Sample collection

During days 44–46 of the experimental period, the feed intake of each goat was recorded daily. Samples of TMR and orts were collected and stored at –20°C until further analysis. The dairy milk yields obtained from all the goats were recorded from day 44 to 46 of the experimental period. Milk samples were collected at 07:00 and 18:00 and mixed at a ratio of 1:1. A 40 mL subsample of milk was transferred to vials preserved with 2-bromo-2-nitropropane-1-3-diol and stored at 4°C for composition analysis. Approximately 80 mL subsamples, without any treatment, were stored in two 50 mL centrifuge tubes at –20°C. Urine and fecal samples were collected daily from days 44 to 46 of the experimental period. The urine was collected in a plastic container containing 200 mL of diluted sulfuric acid which was used to maintain the pH at <3. Urine volumes were recorded daily, with 5% of the urine samples stored in vials. Urine samples from each goat were mixed for three consecutive days and stored at –20°C until nitrogen balance and purine derivative analysis were performed. The total fecal output of each goat was collected into large metal nets that were placed under the metabolic cages and weighed daily. A 5% aliquot of each goat feces was transferred into self-sealed plastic bags daily, mixed for three consecutive days, and stored at –20°C until chemical analysis was performed.

On days 47 and 48 of the experimental period, approximately 1 h after the morning feed, blood samples were collected from the jugular vein using 10 mL vacutainer tubes containing sodium heparin. The tubes were immediately centrifuged at 3,500 × *g* at 4°C for 15 min to separate the plasma (Mendowski et al., 2020). The plasma samples were transferred to 2 mL centrifuge tubes to determine the plasma antioxidant capacity, biochemical parameters, and endocrine indices. On days 49 and 50, rumen fluid samples were collected through the ruminal cannula *via* suction using a hose 1 h after the morning feed (Mendowski et al., 2020). Rumen fluid was filtered through four layers of cheesecloth, and the pH was determined immediately. A 10 mL subsample of ruminal fluid was acidified immediately using 2 mL of metaphosphoric acid (25%, m/v) to determine the VFA, and ammonia-nitrogen (NH_3_-N) contents. A 40 mL subsample without metaphosphoric acid was collected into a 50 mL centrifuge tube and stored at –20°C for ruminal bacterial diversity analysis. All the samples of every goat and data point was included in the analysis.

### Sample analysis

Samples of TMR, orts, and feces were dried at 65°C in a forced-air oven to a constant weight to measure the DM, CP, ether extract, acid detergent fiber (ADF), and neutral detergent fiber (NDF) contents by wet chemistry. The DM was determined using a forced-air oven (AOAC method 930.15). The nitrogen content was determined using a Kjeldahl auto-analyzer (VELP DK20, Usmate, Italy), and the CP was calculated as *N* × 6.25 (AOAC method 990.03). The ether extract content was determined using the AOAC International method 920.39 (AOAC method 920.39). The ADF and NDF contents were measured according to a previously described method (Van Soest et al., 1991). Milk subsamples preserved in 2-bromo-2-nitropropane-1-3-diol were analyzed for true protein, fat, lactose, and total milk solids using infrared spectroscopy (Foss FT 120, Denmark). The nitrogen content in the milk, fecal, and urine samples was determined using the Kjeldahl method (AOAC method 990.39). The NH_3_-N concentration in rumen fluids and urine was measured using a phenol-hypochlorite assay. The rumen AA contents were determined as previously described (Liu et al., 2020). In the rumen fluid preserved with metaphosphoric acid, the VFA content was analyzed using gas chromatography (Agilent 7890A, USA). The allantoin, xanthine, uric acid, and hypoxanthine contents in the urine samples were measured as previously described (Chen and Gomes, 1992).

The total protein, albumin, globulin, creatinine, and β-hydroxybutyric acid content and the activities of alanine and aspartate aminotransferase in the plasma of dairy goats were analyzed using a Beckman AU680 Automatic Biochemical Analyzer (Beckman Coulter Inc., FL, USA). The total AA content, NH_3_ concentration, glutathione peroxidase (GSH-Px), superoxide dismutase (SOD), catalase, and total antioxidant capacity (T-AOC) in plasma were quantified using total AA (A026-1-1), blood ammonia (A086-1), GSH-Px (A005-1), SOD (A001-3-2), catalase (A007-1-1), and T-AOC (A01502-1) assay kits from Nanjing Jiancheng Bioengineering Institute (Nanjing, China), respectively. The levels of growth hormone, prolactin, estradiol, and insulin in plasma were determined using a DFM-96 Gamma Radioimmunoassay Counter (Zhongheng Electromechanical Technology Development Co., Ltd, Hefei, China). Insulin-like growth factor 1 (IGF-1) was determined using a bovine IGF-1 ELISA kit (Shanghai Ketao Biotechnology Co. LTD, Shanghai, China) according to the manufacturer’s instructions.

The total DNA of rumen microorganisms was extracted using the hexadecyltrimethylammonium bromide method and measured using Nanodrop One (Thermo Scientific, MA, USA). The sequencing library was constructed using the MGIEasy kit (BGI, China) and the BGISEQ-500, with read lengths of 2 × 100 base pairs (bp), was used to sequence the libraries. Quality control analysis of the original data was performed using the TrimGalore software to remove low-quality reads with lengths less than 50 bp and average base mass less than 20 (Mehdipour et al., 2020). The relevant data were then compared with the host (sheep, alfalfa, corn, and soybean) database using BM Tagger to remove the host data (Uritskiy et al., 2018). The sequence was then annotated with the species using the Kraken 2 software (Kopylova et al., 2012) and the GTDB-r89 database (Waschulin et al., 2022). The relative abundance of species was analyzed by principal component analysis and the diversity analysis was performed using MicrobiomeAnalyst (Chong et al., 2020). The species composition and community structure of the rumen microbiome were tested for significance using linear discriminant analysis effect size analysis.

### Calculations and statistical analysis

Feed conversion ratio was calculated as previously described (Sears et al., 2020) using the following equation:

Feed conversion ratio (%) = 3.5% FCM/DM intake;

3.5% FCM = [0.4324 × milk yield (kg)] + [16.216 × milk fat yield (kg)],

where FCM = fat-corrected milk; DM = dry matter.

Data were analyzed using the GLM model of SAS v.9.4 (SAS Institute Inc., Cary, NC, USA) as follows:

Y = ijμ+B +iT +jε,ij

where Y_ij_ = dependent variable; μ = overall mean; B_i_ = fixed effect of random block (i = 1 to 5); T_j_ = fixed effect of BCA level (0, 2, or 6 g); and ε_ij_ = experimental error.

Differences among treatments were considered significant at *P* < 0.05, based on Tukey’s multiple comparisons of SAS v.9.4 (SAS Institute Inc., Cary, NC, USA). Results were expressed as least squares means and the means ± standard error of the mean (SEM).

## Results

### Intake, milk yield and composition, and feed conversion ratio

Milk yield in BH was 25.34%, which was significantly greater than that in control group (*P* = 0.04), however no significant difference was observed between the control and BL groups (*P* = 0.70; Table 2). Milk protein and total solid contents were increased significantly in BH by 9.73 and 6.38% (*P* < 0.01), respectively. The feed conversion ratios of dairy goats in BL and BH were increased significantly by 34.92 and 31.75% (*P* < 0.01), respectively. However, body weight, dry matter intake (DMI), milk fat, and lactose contents were not significantly affected by BCA supplementation.

**TABLE 2:** Effects of biochanin A on body weight, dry matter intake, milk yield, and composition.

Items	Treatments			SEM	*P-*value
	Control	BL	BH		
Body weight, kg	66.67	65.32	65.17	1.10	0.860
Dry matter intake, kg/d	1.88	1.45	1.90	0.10	0.119
Milk yield, kg/d	1.46^b^	1.40^b^	1.83^a^	0.076	0.028
Milk composition, %	–	–	–	–	–
Protein	3.64^b^	4.01^ab^	4.32^a^	0.096	0.020
Fat	4.39	4.74	4.73	0.096	0.326
Lactose	4.50	4.59	4.72	0.038	0.072
Total solid	12.54^b^	13.34^ab^	13.76^a^	0.176	0.021
Feed conversion ratio^1^, %	0.63^b^	0.85^a^	0.83^a^	0.030	0.043

^a–b^Different lowercase letters in row represent significant differences (*P* < 0.05).

^1^Feed conversion ratio (%) = 3.5% FCM/dry matter intake; 3.5% FCM = [0.515 × milk yield (kg)] + [13.86 × milk fat yield (kg)].

Means within a row without a common superscript lowercase letter differ significantly (*P* < 0.05, *n* = 10).

### Rumen fermentation parameters

As shown in Table 3, the total VFA, acetate, propionate, butyrate, valerate, and isovalerate contents in the rumen of dairy goats were increased significantly with BCA supplementation (*P* < 0.05). However, the pH and NH_3_-N concentration in the rumen were decreased significantly with BCA supplementation (*P* < 0.05).

**TABLE 3:** Effects of biochanin A on rumen fermentation of dairy goats.

Items	Treatments			SEM	*P*-value
	Control	BL	BH		
pH	6.71^a^	6.23^b^	6.38^b^	0.065	0.011
Total volatile fatty acids, mmol/L	68.7^b^	96.2^a^	91.3^a^	3.05	<0.001
Acetate, mmol/L	44.6^b^	64.6^a^	58.7^a^	2.07	<0.001
Propionate, mmol/L	14.6^b^	20.1^a^	18.6^ab^	0.867	0.037
Butyrate, mmol/L	7.34^b^	9.34^ab^	10.92^a^	0.505	0.009
Isobutyrate	0.60^ab^	0.45^b^	0.73^a^	0.040	0.008
Valerate	0.94^b^	1.08^b^	1.42^a^	0.076	0.026
Isovalerate	0.71^b^	0.64^b^	0.95^a^	0.045	0.006
NH_3_-N, mg/dL	20.4^a^	17.1^b^	16.7^b^	0.484	0.004

Means within a row without a common superscript lowercase letter differ significantly (*P* < 0.05, *n* = 10).

### Antioxidant capacity and endocrine indexes in plasma

The GSH-Px activity in plasma was significantly increased with the increase in BCA treatment (Table 4), with GSH-Px in BL increasing by 14.83% and BH increasing by 44.81% (*P* = 0.02). The T-AOC in BH was significantly higher (*P* = 0.03) than that in BL. However, SOD and catalase activities were not significantly affected by BCA supplementation. Furthermore, the total protein, albumin, globulin, uric acid, creatinine, and β-hydroxybutyric acid contents, in addition to the alanine and aspartate aminotransferase activities, were not significantly affected by BCA supplementation (Table 5).

**TABLE 4:** Effects of biochanin A on antioxidant capacity of plasma.

Items	Treatments			SEM	*P*-value
	Control	BL	BH		
Superoxide dismutase, U/mL	20.71	22.46	19.23	0.649	0.184
Glutathione peroxidase, U/mL	577^b^	662^ab^	836^a^	37.9	0.020
Catalase, U/mL	2.22	2.34	1.86	0.177	0.269
Total antioxidant capacity, mmol/L	0.55^ab^	0.53^b^	0.59^a^	0.011	0.035

Means within a row without a common superscript lowercase letter differ significantly (*P* < 0.05, *n* = 10).

**TABLE 5:** Effects of biochanin A on the changes of plasma biochemical parameters.

Items	Treatments			SEM	*P*-value
	Control	BL	BH		
Total protein, g/L	84.60	84.32	81.86	0.799	0.343
Albumin, g/L	33.40	32.08	33.59	0.490	0.433
Globulin, g/L	51.19	52.25	48.26	0.96	0.232
Alanine aminotransferase, U/L	15.76	16.47	13.36	1.06	0.477
Aspartate aminotransferase, U/L	112	126	89.5	6.96	0.081
Uric acid, μmol/L	0.90	2.69	2.85	0.473	0.127
Creatinine, μmol/L	36.33	33.66	36.40	0.95	0.360
β-hydroxybutyric acid, mmol/L	0.32	0.37	0.34	0.016	0.552

The levels of 17β-estradiol and growth hormone in the plasma of dairy goats in the BH group were significantly higher than those in the BL group (*P* < 0.05; Table 6). The IGF-1 content was significantly increased by BCA supplementation (*P* < 0.05). Compared with control, the IGF-1 content in BL and BH was increased by 28.26 and 26.79%, respectively. However, plasma prolactin and insulin levels were not significantly affected by BCA supplementation.

**TABLE 6:** Effects of biochanin A on endocrine indexes of plasma.

Items	Treatments			SEM	*P*-value
	Control	BL	BH		
17β-estradiol, pg/mL	321^ab^	282^b^	381^a^	14.51	0.003
Prolactin, μIU/mL	72.81	49.82	62.12	4.79	0.208
Growth hormone, ng/mL	1.02^ab^	0.85^b^	1.27^a^	0.072	0.027
Insulin, μIU/mL	18.80	16.79	20.21	0.810	0.114
Insulin-like growth factor 1, ng/mL	140^b^	180^a^	178^a^	6.61	0.020

Means within a row without a common superscript lowercase letter differ significantly (*P* < 0.05, *n* = 10).

### Rumen microbiome

**FIGURE 1:**
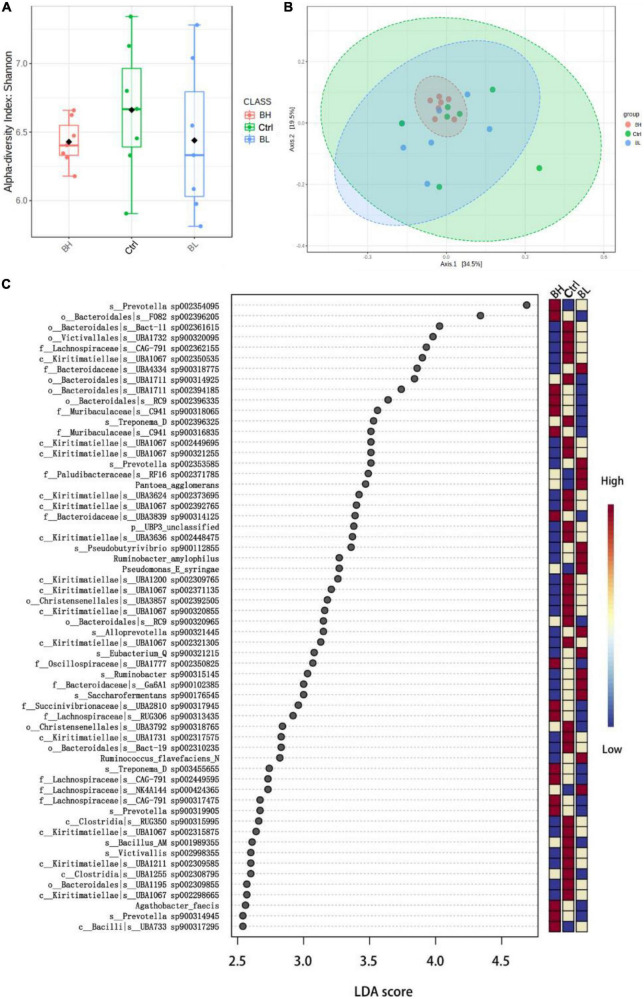
Effect of biochanin A (BCA) on the structure of rumen microbiome of dairy goats (*n* = 7, mean ± SEM). **(A)** Diversity (Shannon index) analysis of rumen microbiome. **(B)** Principal component analysis of rumen microbiome. **(C)** Linear discriminant analysis (LDA) Effect Size analysis of rumen microbial differential flora in genus level. Ctrl, control group; BL, low level BCA group; BH, high level BCA group.

Rumen microbial diversity was not significantly affected by BCA supplementation (Figure 1A). However, the relative abundance of microbial flora was influenced by BCA supplementation (Figure 1B). The quantity of *Verrucomicrobiota* bacteria was significantly decreased, while that of nitrogen metabolism and cellulolytic bacteria was significantly increased, including *Prevotella sp.*, *Treponema sp.*, *Ruminococcus flavefaciens*, and *Ruminobacter amylophilus* (*P* < 0.05; Figure 1C). Thus, BCA regulated the flora of the rumen microbiome, promoting nitrogen metabolic efficiency in the rumen of dairy goats.

## Discussion

Currently, few studies have investigated the influence of BCA on the growth and production performance of dairy goats and cows. Supplementation of steers with BCA (6.3 g/d) exhibited an increase in the average daily gain. The reduction in ammonia concentration was attributed to BCA, which inhibited HAB fermentation of AA deamination (Harlow et al., 2017c). Steers supplemented with 0.91 kg of red clover hay (equivalent to 0.9–1.4 g BCA) and 1.51 kg DDG also exhibited daily gain (Harlow et al., 2020). When the grass silage was replaced with red and white clover silage, the DMI was increased by 1.2 and 1.3 kg, respectively, and the milk yield was increased by 1.5 and 2.2 kg/d, respectively (Steinshamn, 2010). In this study, milk yield and composition were increased when dairy goats were fed BCA. Similarly, supplementation with flavonoid-rich plant extracts improved the milk yield of dairy cows; however, flavonoids from different sources have different effects (Gessner et al., 2015; Zhan et al., 2017). Alfalfa flavonoid extract increased milk lactose synthesis and secretion by reducing the milk fat and protein content (Zhan et al., 2017). However, soy isoflavone supplementation reduced milk production (Kasparovska et al., 2016). These varying results could be caused by extracts from different sources containing different types and amounts of flavonoids. The expression of milk protein and lactose synthesis-related genes were inhibited by BCA and genistein, however, they were upregulated by formononetin and daidzein, and the metabolite of BCA, *p-*ethylphenol (Tsugami et al., 2017).

The nitrogen intake of dairy goats was not significantly affected by BCA addition; however, urine secretion was increased with BCA addition. Urinary nitrogen excretion remained constant. BCA could delay the rate of urea hydrolysis in the rumen, thereby allowing for more urea to be used by rumen microorganisms for microbial protein synthesis. Furthermore, BCA reduced the degradation of proteins and AAs in the rumen by inhibiting the activity of proteolytic bacteria (Liu et al., 2020) and hyper-ammonia-producing bacteria (Harlow et al., 2017c). BCA supplementation increased milk yield, indicating that dietary N utilization efficiency was improved (Harlow et al., 2017c; Pereira et al., 2020). Fewer non-degradable rumen proteins increased protein utilization and produced more milk protein (Morales et al., 2010). In another study, the acetate, propionate, and total VFA contents increased, and pH was decreased by BCA supplementation, which affected cellulose fermentation by rumen cellulolytic bacteria (Harlow et al., 2018). Similar results were obtained when BCA was added to a corn diet fermented by amylolytic bacteria; BCA increased the activity of lactic acid-utilizing bacteria and reduced the starch degradation rate (Harlow et al., 2021). However, the VFA content remained unchanged when no dietary supplements were added to the artificial medium (Liu et al., 2020). In contrast to these studies, all the VFA contents were increased by BCA in this study, which may be caused by different fermentation substrates (Harlow et al., 2020, 2021). In addition, the total VFAs and propionate contents in the rumen were also increased by replacing corn silage with different proportions of red clover (Castro-Montoya et al., 2018). Thus, the increase in VFAs by BCA was dependent on the fermentation substrate.

In this study, rumen ammonia nitrogen concentration was linearly decreased with increasing BCA. The hydrolysis rate of urea was decreased, which ultimately resulted in the inhibition of AA degradation by BCA (Liu et al., 2020). The inhibition of AA degradation by BCA was mainly achieved by inhibiting the growth of hyper-ammonia-producing bacteria, which ferment AAs into ammonia in the rumen (Harlow et al., 2017c). Hyper-ammonia bacterial growth was reduced not inhibited by BCA through the enhancement, by rumen microorganisms, of antibacterial activity of bacteriocin, which reduced the ammonia nitrogen content rather than exerting an inhibitory effect, thereby indicating that BCA had synergistic antibacterial activity (Flythe et al., 2013). Crude red clover phenolic extracts also inhibited the growth of hyper-ammonia-producing bacteria (*Clostridium sticklandii SR*) (Kagan and Flythe, 2012), increased bypass protein, and promoted the by reducing protein nitrogen loss (Harlow et al., 2017c). Therefore, BCA promoted growth of steers rumen fermentation and increased microbial protein production. In addition, the SOD, GSH-Px, and catalase are the main antioxidant enzymes in animals. Serum GSH-Px activity reflected oxidative stress and contributed to oxidative defense in animals (Tuzun et al., 2002). The activities of SOD and GSH-Px were enhanced by BCA through inhibiting nicotinamide adenine dinucleotide phosphate and malondialdehyde production (Yu et al., 2019). In addition, BCA prevented oxidative stress by enhancing the total antioxidant status and the levels of SOD and catalase (Jalaludeen et al., 2016; Sadri et al., 2017). Isoflavone BCA is activating a novel nuclear factor erythroid 2-related factor 2-antioxidant response element activator and can protect against oxidative damage (Yu et al., 2019). Flavonoid extracts containing BCA had a similar effect on improving the antioxidant capacity of livestock and poultry. In chickens supplemented with soybean isoflavone, increased T-AOC and SOD levels in the plasma were reported (Jiang et al., 2007). Dietary supplementation with flavonoids from *Scutellaria baicalensis Georgi* enhanced the antioxidative ability of broilers (Liao et al., 2018). The activities of SOD and GSH-Px were also increased by feeding alfalfa flavone extract to dairy cows (Zhan et al., 2017). The GSH-Px activity was increased with an increase in BCA supplementation, indicating that BCA can improve the antioxidant performance of dairy goats by enhancing T-AOC and the activities of antioxidant enzymes.

Plasma biochemical parameters reflected the metabolism of nutrients and health conditions of the animal body (Wang et al., 2011). No differences were observed in the total protein, albumin, globulin, uric acid, creatinine, and β-hydroxybutyric acid contents, suggesting that BCA had no effect on the health of dairy goats. Alanine and aspartate aminotransferase are vital indices of liver function, reflecting the permeability and metabolic function of liver cells (Li et al., 2017). Increases in alanine and aspartate aminotransferase activities were caused by damage to the integrity of the hepatic membrane architecture (Kotyzova et al., 2013). In this study, although there is no significant difference, aspartate aminotransferase activity had a decreased trend in the BH group, indicating that a high dose of BCA may have a hepatoprotective effect in dairy goats, and this trend was also observed in a previous study in rats (Breikaa et al., 2013). PUN and NH_3_-N were the final protein metabolites that reflected AA metabolism (Li et al., 2017). PUN concentrations reached a peak approximately 2 h after feeding, then gradually decreased, reverting to normal levels at 6 h (Sinclair et al., 2012). A similar trend was observed in the NH_3_-N concentrations, which may have resulted from the decreased hepatic clearance of NH_3_-N (Ludden et al., 2000). Soybean isoflavone, an isoflavone phytoestrogen, is a physiological regulator of the reproductive and nutritional processes of the body through the neuroendocrine system to improve the production performance of animals (Jiang et al., 2007). Isoflavone promoted the development of mammary glands, increased milk yield, and accelerated animal growth by inducing changes in endogenous hormone levels in blood, such as prolactin, growth hormone, and IGF-1 (Tsugami et al., 2017). In this study, BCA treatment significantly increased the levels of 17β-estradiol, growth hormone, and IGF-1. Similarly, the levels of 17β-estradiol, growth hormone, and prolactin in the plasma of mid-lactation dairy cows were also increased by soybean isoflavone daidzein (Tsugami et al., 2017). These hormones can regulate the lactation of dairy goats and the structural development of mammary gland, and estradiol and IGF-1 can regulate mammary epithelial cells by binding to estrogen receptor and maintaining its expression (Tsugami et al., 2017). However, there are limited reports on the effects of BCA on the endocrine system of animals; therefore, further studies are required to explore the underlying mechanisms.

In this study, the flora structure of the rumen microbiome was modulated by BCA supplementation, which promoted nitrogen metabolic efficiency in the rumen. Similarly, in another study, the abundance of Bacteroidetes was increased by isoflavone-enriched feed, resulting in a high nitrogen metabolic efficiency in the rumen (Kasparovska et al., 2016). Rumen microbial diversity was not significantly affected, whereas the abundance of rumen microbiota was significantly affected by red clover isoflavones (Melchior et al., 2018). These results indicated that the nitrogen metabolic efficiency in the rumen was improved by BCA treatment by modulating the structure of the rumen microbiome. In this study, the flora of nitrogen metabolism and cellulolytic bacteria in rumen was regulated by BCA, and these rumen microbiota can promote nitrogen metabolic efficiency of dairy goats, resulting in enhancing feed conversion and increasing VFA production. As we know, acetate and butyrate are the precursors of milk fat, and propionate is the precursor of lactose in milk. Therefore, the increased VFA led to the improvement in milk production. In addition, reduced ammonia can be used by rumen microbes, and the increase of microbial protein can increase the milk protein, also leading to the increased feed conversion and VFA production. Additionally, in health dairy goats, the improvement in antioxidative capacity can enhance their resistance of pathogen and diseases, which is beneficial to the health of goats. Moreover, unchanged plasma biochemical parameters indicated that BCA had no side effects on the immune and health of dairy goats. The altered endocrine indexes indicated BCA can increased the production performance of dairy goat through hormone to some extent, including 17β-estradiol, GH, and IGF-1.

## Conclusion

Milk production performance, nitrogen metabolism, and the feed conversion ratio of dairy goats were improved by BCA supplementation. Rumen AA degradation were inhibited, whereas VFAs were increased with BCA supplementation, indicating that rumen fermentation was promoted. Antioxidant performance (GSH-Px activity and T-AOC) and endocrine hormones levels (prolactin, growth hormone, and IGF-1) were also increased by BCA supplementation. The relative abundance of rumen microbiota were also affected by BCA supplementation, such as decreased *Verrucomicrobiota* and increased nitrogen metabolism and cellulolytic bacteria, including *Prevotella sp*., *Treponema sp*., *Ruminococcus flavefaciens*, and *Ruminobacter amylophilus*. Thus, dairy goat supplemented with BCA at a concentration of 6 g/day per goat can improve its performance and health status.

## Data availability statement

The original contributions presented in this study are publicly available. This data can be found here: https://nmdc.cn/resource/genomics/project/detail/NMDC10018266.

## Ethics statement

This animal study was reviewed and approved by Animal Care and Use Committee of the Institute of Animal Science of the Chinese Academy of Agricultural Sciences (approval no. IAS2020-97).

## Author contributions

YL, JW, and SZ conceived and designed the experiments. QX and YL wrote and prepared the original draft. QX, WD, NZ, JW, and SZ edited the manuscript. QX, JW, and SZ critically reviewed the manuscript. All authors reviewed and approved the final manuscript.
